# Effect of Heat Treatment on the Microstructure and Mechanical Properties of the Al_0__.6_CoCrFeNi High-Entropy Alloy

**DOI:** 10.3390/ma16227161

**Published:** 2023-11-14

**Authors:** Pengyu Hou, Yue Yang, Leilei Zhang, Yi Meng, Yan Cui, Leigang Cao

**Affiliations:** Department of Materials Science and Engineering, North China University of Technology, Beijing 100144, China; pengyu5hpy@163.com (P.H.); yyue@ncut.edu.cn (Y.Y.); zhangleilei@ncut.edu.cn (L.Z.); mengyi@ncut.edu.cn (Y.M.); cuiyan@ncut.edu.cn (Y.C.)

**Keywords:** high-entropy alloy, phase transition, strengthening, microstructure, mechanical property

## Abstract

The effect of heat treatment on the microstructure and tensile properties of an as-cast Al_0.6_CoCrFeNi high-entropy alloy (HEA) was investigated in this paper. The results show that the as-cast Al_0.6_CoCrFeNi HEA presents a typical FCC dendrite morphology with the interdendritic region consisting of BCC/B2 structure and heat treatment can strongly affect the microstructure and mechanical properties of HEA. Microstructure analysis revealed the precipitation of a nano-sized L1_2_ phase in the FCC dendrite and the formation of the FCC and *σ* phases in the interdendritic region after annealing at 700 °C. The coarse B2 phase was directly precipitated from the FCC dendrite in the 900 °C-annealed sample, with the coexistence of the B2, FCC, and σ phases in the interdendritic region. Then, the interdendritic region converted to a B2 and FCC dual-phase structure caused by the re-decomposition of the *σ* phase after annealing at 1100 °C. The tensile test results show that the 700 °C-annealed HEA presents the most significant strengthening effect, with increments of corresponding yield strength being about 107%, which can be attributed to the numerous nano-sized L1_2_ precipitates in the FCC dendrite. The mechanical properties of 1100 °C-annealed alloy revert to a level close to that of the as-cast alloy, which can be attributed to the coarsening mechanism of B2 precipitates and the formation of a soft FCC phase in the interdendritic region. The observed variation in mechanical properties during heat treatment follows the traditional trade-off relationship between strength and plasticity.

## 1. Introduction

High-entropy alloys (HEAs), since being defined in 2004 [1,2], have been paid increasingly intensive attention for their potential engineering applications due to their excellent mechanical properties. In many cases, multi-component HEAs with a key feature of high configurational entropy tend to form simple crystal structures such as FCC, BCC, HCP, or a mixture of two or more phases [3,4]. Until now, many efforts have been carried out to optimize the elemental constitution and microstructure for advanced HEAs with high strength and high ductility. In this process, more and more investigations have revealed the effectiveness of the classical strengthening mechanisms via the formation of solid solution, the dispersive second phase, grain-refined microstructure and even deformation-induced phase transition [5,6,7].

In fact, a remarkable strengthening effect has been achieved on low-strength FCC-structured HEAs using microstructure tailoring due to their good ductility [8]. For this purpose, severe plastic deformation methods are usually chosen, such as cold rolling, high-pressure torsion, and equal channel angular pressing, which might also incorporate subsequent annealing treatment [8]. For example, the remarkable reduction in grain diameter from 100 μm to about 50 nanometers in CoCrFeNiMn HEA by means of high-pressure torsion results in a significant increase in strength, being 1950 MPa [9].

Actually, if there are phase transition behaviors in such thermal mechanical processes, the mechanism of microstructure evolution and its effect on mechanical properties will become more complex. An example is Al*_x_*CoCrFeNi HEAs [10], which are a promising and important engineering material. The corresponding as-cast microstructures, as well as the associated unstable phases, are very sensitive to Al content. Al*_x_*CoCrFeNi HEAs with low Al content (*x* ≤ 0.45) are usually solidified into a simple FCC structure [11]. With the increase in Al content, the BCC phase can solidify directly in the interdendritic (ID) region of the as-cast Al_0.5_CoCrFeNi HEA, with the fraction of the ID region being 10.6% [12]. Although the Al_0.6_CoCrFeNi HEA also presents a dendrite morphology, the ID region solidified into a BCC/B2 spinodal nano-structure, and its fraction increases to about 27.5% according to the observations in the present paper. Furthermore, the Al_0.7_CoCrFeNi HEA solidifies into a eutectic-like morphology, comprising alternating structures of FCC single-phase and spinodal BCC/B2 phases, giving it a promising strength–ductility balance [13]. When x exceeds 0.8, the spinodal structure will be predominant [14,15,16].

It has been proved that the thermal-mechanical processing is one of the most promising routes to improve the mechanical properties of low-Al Al*_x_*CoCrFeNi HEAs with excellent tensile strength and plasticity. Significant grain refinement (with grain size varying from 5.7 μm to 144 μm) has been successfully achieved in Al_0.3_CoCrFeNi HEAs using cold rolling (90%) and subsequent annealing, presenting a strong Hall–Petch relationship for both the hardness and the tensile yield strength [5]. The enhanced strength can be attributed to the refined FCC grains, B2 phase and even *σ* intermetallic phase. The latter two phases precipitate from the FCC phase during the annealing process at intermediate temperatures of 550–900 °C. Similarly, Al_0.7_CoCrFeNi HEA can also be strengthened due to the massive FCC-BCC interfaces and the L1_2_ precipitates in the FCC phase in the thermal-mechanical processing [13]. Although the thermal-mechanical processing can also affect the mechanical properties of high-Al Al*_x_*CoCrFeNi HEAs (*x* ≥ 0.8), the corresponding assessment is mainly carried out on the basis of the hardness test and compressive test due to the related brittle feature, the results of which are not stated in this paper [15,16].

The thermal stability analysis revealed that the supersaturated FCC phase, primarily, solidified from the melt, and the disordered BCC phase was unstable, both of which will undergo solid-state phase transitions at an elevated temperature and, thus, influence the microstructure and mechanical properties [17,18]. It is worth noting that the defects induced via pre-deformation in the thermal-mechanical processing could strongly facilitate the nucleation and growth process during the phase transition [19]. This indicates that the microstructure evolution caused by solid-state phase transition might be different in an individual annealing process, which might be obscured by the accelerating effect caused by the thermal-mechanical process. Therefore, in the present paper, phase transformation behaviors of as-cast Al_0.6_CoCrFeNi HEA under different treatment conditions and their effects on phase constitution, microstructure evolution, and resulting mechanical properties were investigated. Based on a similar experimental design, the Al_0.5_CoCrFeNi HEA was significantly strengthened after annealing at 850 °C, which was mainly attributed to the precipitation behavior in the FCC dendrite, while the strengthening effect with a slight increase of 3.8% in the BCC phase caused by potential BCC-FCC phase transition, pending direct observations, should be relatively minor. That is to say, the phase transition characteristics corresponding to FCC dendrite and BCC interdendritic regions are inadequate [18]. Instead, for the Al_0.6_CoCrFeNi HEA, the fraction of the interdendritic region with a BCC/B2 spinodal structure reaches to approximately 27.5%, which provides a proper opportunity to investigate the coupling effect of different phase transitions that occurred in the DR and ID regions on the microstructure and mechanical properties of the Al_0.6_CoCrFeNi HEA. Additionally, the research has potential application prospects for the heat-treatable HEAs with modified microstructure assisted via directional temperature field [20], electromagnetic field [21] and so on.

## 2. Materials and Methods

The Al_0.6_CoCrFeNi ingots were prepared using an electric arc-melting furnace with a water-cooled copper hearth (MTDH-600; Shanghai Mengting Instrument Equipment Co., Ltd., Shanghai, China). The purity of the constituent metals was higher than 99.99%. The furnace was evacuated to a pressure of 3 × 10^−3^ Pa using a rotary pump and a molecular pump and then back-filled with argon (repeated five times) to obtain an Ar (3 × 10^3^ Pa) protective condition before the melting process. The ingots were turned over and melted five times to ensure chemical homogeneity with a maximum heating current of 300 A. Finally, the ingots were placed onto the casting mold and solidified into rods measuring 10 mm × 10 mm × 60 mm using the drop-casting method [7].

The casting rods were annealed at temperatures of 700 °C, 900 °C, and 1100 °C, respectively, for 3 h and cooled in air. The annealed samples for microstructure analysis were mounted (160 °C) using conductive resin and then ground progressively using a series of abrasive SiC papers down to 2000 grit and polished using 5 μm, 2.5 μm, 1 μm, and 0.25 μm diamond paste and, lastly, colloidal silica. Ultrasonic cleaning was used to remove the potential residue and optical microscopy was used to check the surface quality at each grinding and polishing stage. The samples were washed using dilute detergent and absolute ethyl alcohol and then dried using hot air between each polishing step. The alloy samples for phase identification were only ground down to 2000 grit, without mounting and polishing procedures.

The microstructure and fracture morphology of the samples were studied by using a scanning electron microscope equipped with a backscattered electron detector (SEM; Zeiss Sigma-300, Carl Zeiss, Cambridge, UK). The elemental composition of the polished samples was also measured with a Bruker Quantax spectrometer system (XFlash detector, Bruker, Berlin, Germany) inside the SEM. A FEI Tecnai G2 F30 transmission electron microscope (TEM, FEI company, Hillsboro, OR, USA) was also used for further detailed microstructure investigation. TEM specimens were fabricated using a Gatan 691 (Gatan, Pleasanton, CA, USA) ion polishing system (as-cast and 1100 °C-annealed specimens) and by using a focused ion beam technology (alloys were annealed at 700 °C and 900 °C) based on the corresponding microstructural complexity.

In situ XRD was conducted to identify the phase constitution of the sample related to the potential thermal phase transformations of the metastable phases (Cu-Kα radiation, SmartLab, Rigaku, Tokyo, Japan). In situ heating was carried out in a sealed chamber under a protective argon atmosphere, in which the sample could be heated to a maximum of 1150 °C. The sample was scanned via X-ray at 25 °C, 700 °C, 900 °C, 1000 °C, and 1100 °C in the heating stage and at 700 °C and 25 °C again in the cooling stage. The sample was kept for 10 min at each set temperature before scanning.

In most cases, the specimens used for tensile tests related to HEAs are usually small due to the limitation of sample size. The thickness of the tensile specimens is usually 1~1.5 mm, according to the literature. However, the corresponding gauge length and width vary greatly, such as 15 mm and 5 mm [6], 14 mm and 2 mm [12], 12.5 mm and 3.2 mm [22], 10 mm and 4 mm [23], 5 mm and 2.5 mm [24], and so on. In the present paper, the standard used for the tensile test is GB/T 228.1-2010 [25], which was released by the National Standard of the PRC. The specimen geometry refers to the equation of *L* = *L*_0_ + 2*b*_0_, where *L*_0_ is the gauge length, and *L* and *b*_0_ are the length and width of the parallel section, respectively. Therefore, tensile samples with an 18 mm long parallel section that has a width of 4 mm and an approximate thickness of 1.1 mm were prepared using an electrical discharge machine. The surfaces of the specimens were ground using SiC paper down to 2000 grit to remove the surface damage. Tensile tests were carried out on an MTS Exceed E44 electronic universal test system (MTS Systems Corporation, Eden Prairie, MN, USA) with a strain rate of 1 × 10^−3^ s^−1^ at room temperature. At least three independent tensile tests were performed on the four different alloys studied to ensure consistency. The deformation of the samples under loading was recorded using an EAG-010M-1000-S extensometer (Reliant Technology LLC., Colorado Springs, CO, USA) with a gauge length of 10 mm and the maximum measurement range of 100%.

## 3. Results

### 3.1. Microstructures of as-Cast Al_0.6_CoCrFeNi Alloy

**Figure 1:**
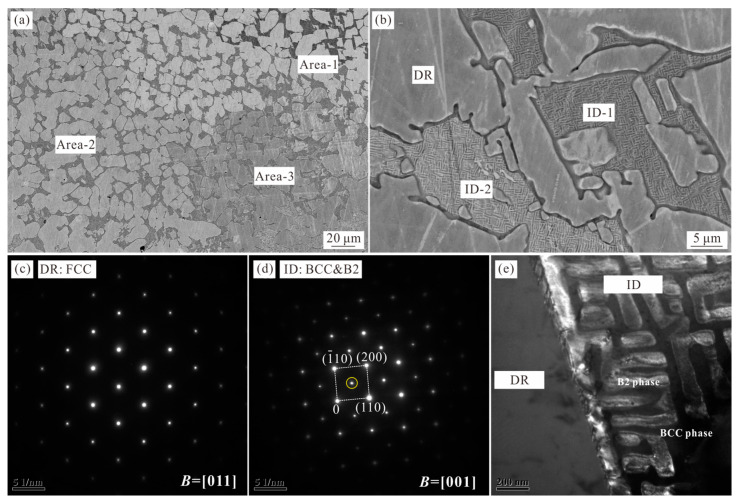
Images (**a**,**b**) show SEM-BSE and TEM dark-field micrographs of as-cast Al_0.6_CoCrFeNi HEA, respectively, showing the typical dendrite morphology. (**c**) SADP of the dendrite, indicating the primary solidification of the FCC phase. (**d**) SADP of the interdendritic region, showing the disordered BCC and ordered B2 structure, respectively. (**e**) The dark field TEM image was taken from the additional spot of the BCC/B2 reflections (yellow circle in (**d**)).

Figure 1 shows the microstructure of as-cast Al_0.6_CoCrFeNi alloy observed using SEM and TEM instruments. It can be seen that the as-cast microstructure presents a typical dendrite structure (DR), with the interdendritic region (ID) presenting a dual-phase morphology (Figure 1b). The BSE image of Figure 1a reveals the contrast difference between the three areas, which were labeled as Area-1, Area-2, and Area-3. The EDX measurement was carried out to analyze the average elemental compositions of these three areas based on the data obtained from five different positions of the sample, the results of which are provided in Table 1. It is clear that the composition of the prepared alloy is equal to the nominal values. Meanwhile, the measured elemental compositions of the dendrite and interdendritic regions from Areas 1–3 shown in Figure 1a are given in Table 2. The results show that the elemental compositions of the ID region of Areas 1–3 are very close, while the elemental compositions of the DR region of Areas 1–3 fluctuate, especially for Co and Cr elements. Overall, the Al element is enriched in the ID region, as it is a BCC stabilizer.

**Figure 2:**
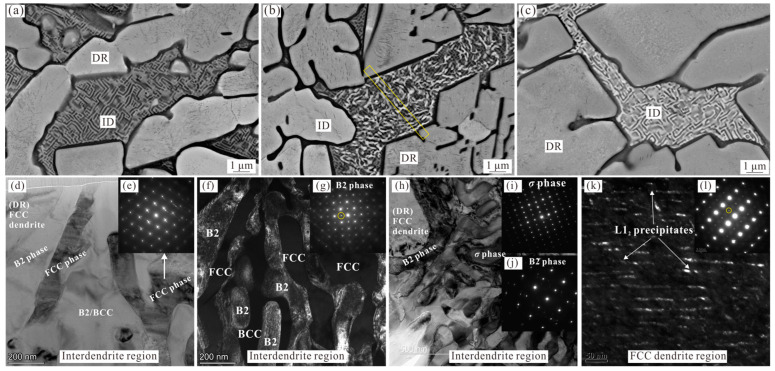
Images (**a**–**c**) show SEM-BSE micrographs of 700 °C-annealed Al_0.6_CoCrFeNi HEA, presenting the three typical morphologies of microstructure evolution in the ID region; (**d**) TEM bright-field image of the ID region labeled by yellow rectangle in (**b**); (**e**) SADP from the [011] zone indicating the bright phase in (**b**) is the decomposed FCC phase; (**f**) the dark field TEM image taken from the additional spot of the BCC/B2 reflections (yellow circle in (**g**)); (**g**) SADP of the B2/BCC phases, showing the diffraction of super-lattice spots; (**h**) TEM bright-field image of the ID region shown in (**c**); (**i**,**j**) SADP of the *σ* and B2 phase, respectively; (**k**) the dark field TEM image taken from the additional spot of the FCC/L1_2_ reflections (yellow circle in (**l**)), showing the precipitation of the L1_2_ phase; (**l**) SADP of the dendrite region, showing the diffraction of super-lattice spots.

Figure 1b was taken from Area-3, indicating the difference in contrast between the ID-1 and ID-2 areas. TEM analysis revealed that the microstructures of ID-1 and ID-2 are the same, with the results of ID-1 shown in Figure 1c–e. TEM-selected area diffraction patterns (SADPs) confirm that the dendrite is a disordered FCC phase and the interdendrite consists of disordered BCC and ordered BCC (B2) phases. Figure 1e shows the dark field TEM image taken from the additional spot of the BCC/B2 reflections (yellow circle in Figure 2d). It can be seen that these additional spots were attributed to the relatively bright areas, corresponding to the ordered B2 phase, and the other constituent phase should be the disordered BCC phase. A similar microstructure with the same phase constitution has been reported in the Al-Co-Cr-Fe-Ni alloy systems [14,26]. The addition of the Al element in the FCC-structured CoCrFeNi high-entropy alloy facilitates the formation of the phases with the body-centered cubic crystal structure. Here, the solidification path of the Al_0.6_CoCrFeNi alloy is for the primary growth of the FCC phase, with the interdendritic region converting to the BCC and B2 phases via a spinodal decomposition.

### 3.2. Microstructure of Annealed Al_0.6_CoCrFeNi Alloy

**Figure 3:**
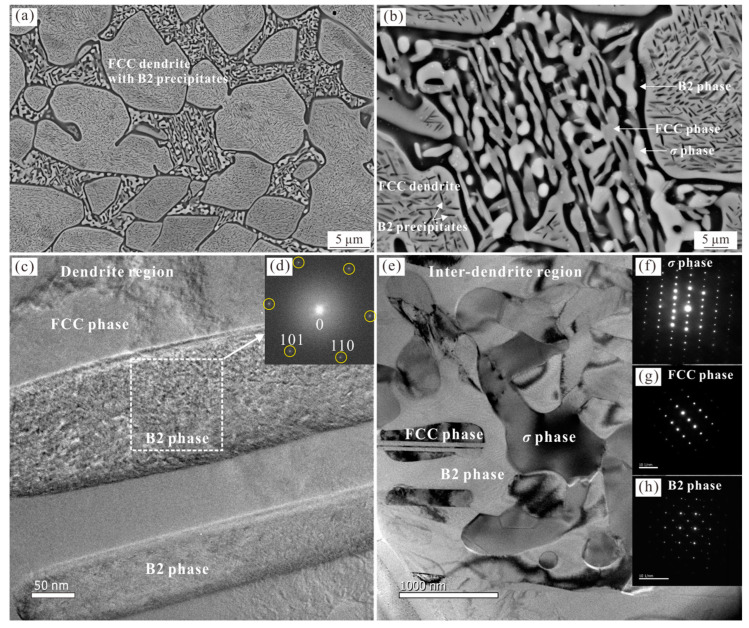
Images (**a**,**b**) show low- and high-magnified SEM-BSE micrographs of the 900 °C-annealed Al_0.6_CoCrFeNi HEA, showing the rod-like precipitation in the dendrite region and a ternary-phase morphology in the interdendritic region. (**c**) High-resolution TEM micrograph of the rod-like precipitation. (**d**) Fourier transform image indicating the rod-like precipitation is B2 phase. The diffraction spots of {110} crystal plane are labeled by yellow circles. (**e**) Bright-field image of the interdendritic region with corresponding SADP patterns (**f**–**h**) showing the existence of the B2 phase, *σ* phase, and FCC phase.

**Figure 4:**
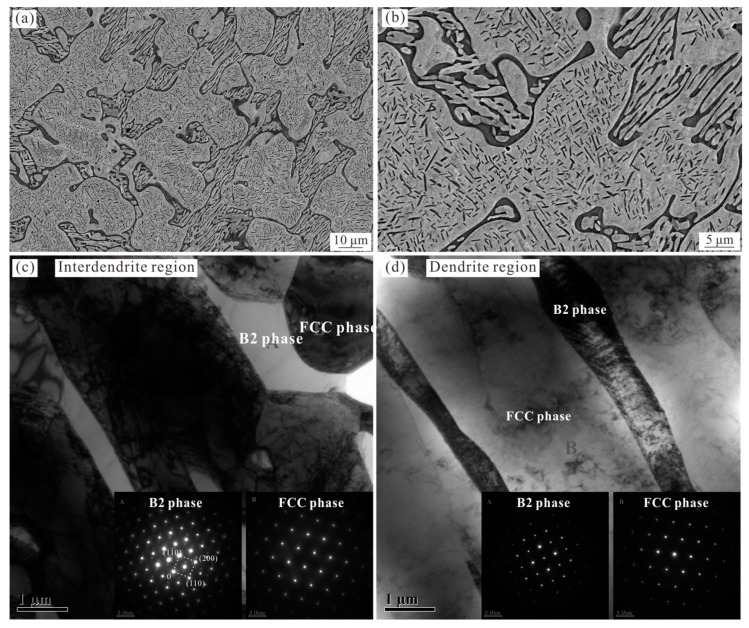
Images (**a**,**b**) show low- and high-magnification SEM-BSE micrographs of the 1100 °C-annealed Al_0.6_CoCrFeNi HEA, respectively, showing the coarse B2 precipitation in the dendrite region and a new dual-phase structure in the interdendritic region. (**c**) TEM image of the interdendritic region, with SADP patterns showing the B2 phase (dark phase in (**b**)) and the FCC phase (gray phase in (**b**)). (**d**) TEM image of the dendrite region, with corresponding SADP patterns showing the distribution of B2 precipitates in the FCC dendrite.

In order to reveal the microstructure evolution via solid-state phase transitions at elevated temperatures, the microstructures of the Al_0.6_CoCrFeNi alloys annealed at 700 °C, 900 °C, and 1100 °C for 3 h were analyzed via SEM and TEM, the results of which are shown in Figure 2, Figure 3 and Figure 4, respectively. In order to confirm the origin of the microstructural evolution, FIB was used to prepare TEM specimens from interdendritic regions of the alloys heat-treated at 700 °C and 900 °C. Although the annealed Al_0.6_CoCrFeNi HEA maintains the same dendrite morphology, these data present two important observations corresponding to the dendrite region and the interdendritic region, respectively.

The SEM and TEM micrographs from the 700 °C-annealed Al_0.6_CoCrFeNi HEA, presented in Figure 2, show three typical morphologies of microstructure evolution in the interdendritic region according to the enhanced contrast of the constituent phases observed using a backscatter SEM detector. Comparing Figure 1b and Figure 2a, it seems that in some regions the phase transition has not yet begun, while the brightness variation presented in Figure 2b,c indicates the occurrence of the phase transition and the formation of additional phases. This result is reasonable since the annealing temperature is slightly higher than the initial decomposition temperature of the BCC phase (about 650 °C). In order to confirm the origin of the observed microstructure, FIB was used to prepare TEM specimens from the dendrite and interdendritic boundary of the structure shown in Figure 2b,c, respectively. Figure 2d,f show the bright field and dark field TEM images of the ID region shown in Figure 2b, respectively. The SADP from the [011] zone presented in Figure 2e confirms that the bright phase in Figure 2b is the FCC phase, which is one of the products of BCC decomposition. The SADP from the area next to the FCC phase of the ID region displays super-lattice spots (Figure 2g) from the ordered B2 phase, which cannot be used to distinguish the existence of the disordered BCC phase from the identical lattice parameters of the BCC and B2 phases. Therefore, the dark field TEM image was obtained, taken from the additional spot of the BCC/B2 reflections (yellow circle in Figure 2g), as shown in Figure 2f. It can be seen that these additional spots were attributed to the relatively bright areas, corresponding to the ordered B2 phase, and the other constituent phase of this area should be the disordered BCC phase. Figure 2h presents the microstructure of the ID region of Figure 2c. Interestingly, the corresponding SADPs (Figure 2i,j) only identify the existence of B2 (ordered BCC phase) and *σ* phases.

In addition, the microstructure of the FCC dendrite region presented in Figure 2a–c also revealed the precipitation of a large number of nano-sized phases in the 700 °C-annealed Al_0.6_CoCrFeNi HEA. Figure 2l shows the [-112] zone axis from the FCC dendrite region, with the additional spots of extra {110} super-lattice reflections in comparison with that of the as-cast alloy (Figure 1c), confirming the presence of the L1_2_ phase. Figure 2k shows the dark field TEM image obtained from the {110} superlattice reflection, clearly revealing that these nano-phases within the FCC dendrite are L1_2_ precipitates.

Figure 3 shows the microstructure of the 900 °C-annealed Al_0.6_CoCrFeNi HEA, presenting a more obvious microstructure evolution. As can be seen in Figure 3a,b, a large fraction of the rod-like phase precipitated from the dendrite. Moreover, the enhanced contrast of the constituent phases in the interdendritic region clearly indicates the existence of three different phases, namely, the dark matrix phase, the gray phase and the spherical bright phase. FIB was also used to prepare TEM specimens from the dendrite and interdendritic boundary to confirm the origin of the observed microstructure. Figure 3c shows a high-resolution TEM image of the rod-like precipitates within the FCC dendrite, with the corresponding Fourier transform image shown in Figure 3d, indicating a body-centered cubic structure. With a further consideration of the thermodynamic calculation [27] and the subsequent results of 1100 °C-annealed samples, these rod-like precipitates were confirmed as ordered B2 phase precipitates, rather than the disordered BCC phase or the L1_2_ precipitates in the 700 °C-annealed sample. The width of the B2 nano-precipitates can reach 100 nm, which is much larger than that of the L1_2_ nano-precipitates in the 700 °C-annealed alloy (Figure 2k). Figure 3e shows the microstructure of the ID region, where the constituting phases are successfully identified as the B2, FCC, and *σ* phases, respectively, according to the corresponding SADPs (Figure 3f–h). This result is consistent with the observations that the disordered BCC phase will convert to B2 and FCC phases when the temperature is above 650 °C [15].

Figure 4 shows the microstructure of the 1100 °C-annealed Al_0.6_CoCrFeNi HΕA analyzed via SEM and TEM, wherein the interdendritic region further converts to a dual-phase structure (Figure 4a,b). TEM analysis confirmed that the dark phase and gray phase are the B2 phase and FCC phase, as shown in Figure 4c. This result is consistent with the fact that the *σ* phase is also unstable and will convert to B2 and FCC phases for temperatures in excess of 950 °C [15,26]. Therefore, the 1100 °C-annealed Al_0.6_CoCrFeNi alloy only consists of the B2 and FCC phases. This result is also consistent with the density variation in the greyscale BSE image which presents only two distinct phases (Figure 4a,b). Meanwhile, the B2 precipitates in the FCC dendrite are coarsened, with the width being about 1 μm (Figure 4d).

The FCC phase in the dendrite region and the B2 phase in the interdendritic region of the 1100 °C-annealed HEA were measured by point-scanning of five random positions, the results of which are provided in Table 3. EDX results show that, after the decomposition reaction, Al and Ni elements of the FCC phase in the dendrite region decreases to 7.9 at.% and 17.1 at.%, respectively. Co, Cr and Fe elements mainly accumulate in the FCC phase, especially for Cr elements, which increases from 21.6 at.% to 26.6 at.%. Conversely, Al and Ni elements mainly accumulate in the B2 phase, being about 31.7 at.% and 30.1 at.%, respectively.

### 3.3. In Situ XRD Analysis of Al_0.6_CoCrFeNi Alloy

**Figure 5:**
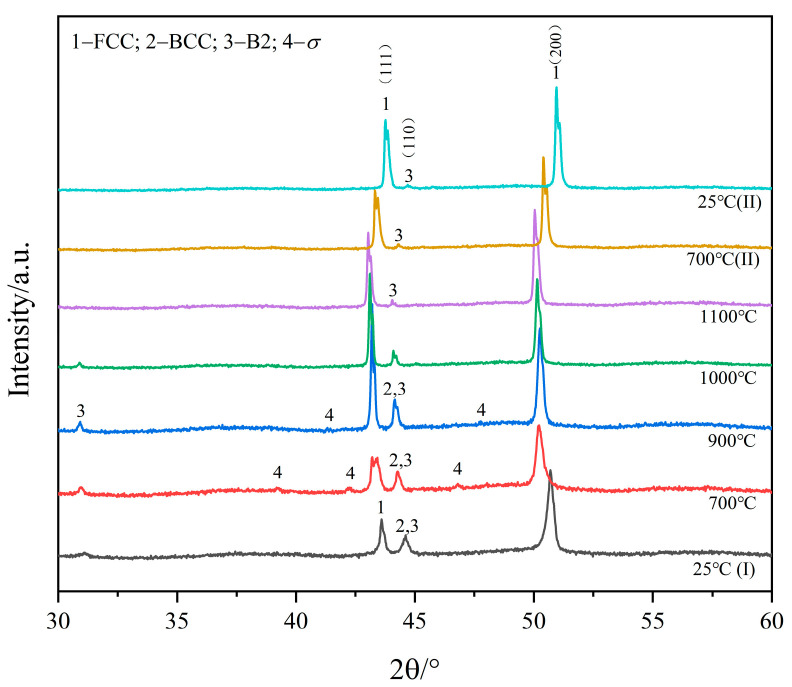
XRD results of in situ heating of the as-cast Al_0.6_CoCrFeNi alloy from about 25 °C to 1100 °C. The heating temperature covers the phase transition relating to the decomposition of the BCC phase and the formation and re-decomposition of the *σ* phase.

Figure 5 shows the results of the in situ XRD heating experiment of the Al_0.6_CoCrFeNi HEA. Comparing the XRD patterns of the alloys before and after heat treatment at room temperature (25 °C) and referring to the results of microstructure analysis, the results indicate that the disordered BCC phase is unstable and converted fully to an ordered B2 and FCC phase. Meanwhile, the decreasing diffraction peaks of the BCC phase (labeled 2 in Figure 5) as the temperature is increased from 900 °C to 1100 °C indicate the gradual decomposition of the *σ* phase. Similar behaviors were also observed in the heat treatment experiments of the AlCrFeNiMo_0.2_ HEA [28]. The relative intensity of the diffraction peak for the B2 and BCC phases significantly decreased after annealing at 1100 °C. The existence of the *σ* phase can also be proved by XRD patterns of the alloys heated at 700 °C and 900 °C according to the corresponding weak diffraction peaks. The corresponding evolution of the *σ* phase is illustrated in Figure 2, Figure 3 and Figure 4. The X-ray diffraction pattern can be used effectively for phase identification as per Bragg’s law, *nλ* = 2*dsinθ*, where *d* is the distance between diffraction planes, *λ* is the wavelength of the X-ray beam, *θ* is the incident/diffracted angle of the beam and *n* is an integer. Here, the predominant constituting phases (FCC, BCC, and B2 phases) are cubic crystal structures. The expected expansion of the crystal lattice during the heating process can give rise to an increase in the distances between diffraction planes (*d*). Therefore, the corresponding diffraction peaks shifted to low diffraction angles during the heating process. By analogy, the diffraction peaks shifted back to the high diffraction angles due to the contraction of the crystal lattice during the cooling process.

### 3.4. Mechanical Properties of as-Cast and Annealed Al_0.6_CoCrFeNi Alloy

**Figure 6:**
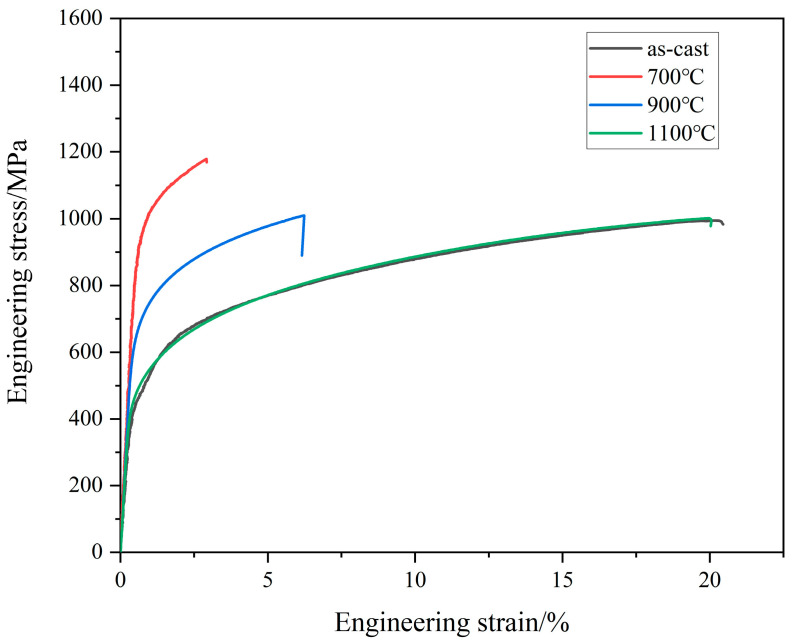
Room-temperature tensile stress–strain curves for the as-cast and annealed Al_0.6_CoCrFeNi HEA.

Figure 6 shows the engineering stress–strain curves for the as-cast and annealed Al_0.6_CoCrFeNi HEAs. According to Figure 6, the yield strength and tensile strength of the as-cast Al_0.6_CoCrFeNi HEAs are 462 MPa and 994 MPa, respectively, which are higher than those of the as-cast Al_0.5_CoCrFeNi HEAs, being 402 MPa and 568 MPa, respectively [29]. This is because the addition of the Al element in the FCC-structured CoCrFeNi HEAs facilitates the formation of the hard BCC phases, giving rise to continuous improvement in the mechanical properties. Meanwhile, the as-cast Al_0.6_CoCrFeNi HEA processes good plasticity with the elongation being 20.3%, which is due to the matrix of the soft FCC phase in the as-cast structure.

The strengthening effect of the annealed HEA is closely related to the characteristics of the reinforcement, where small and hard precipitates with a high fraction and relatively uniform distribution are preferred. As can be seen in Figure 2k, after annealing at 700 °C, a high fraction of the nano-sized L1_2_ phase precipitates from the FCC dendrite region with uniform distribution. This should be the main reason for the significant increase in the yield and tensile strength, being 961 MPa and 1179 MPa, respectively. In addition, it is clear by comparing Figure 1 and Figure 2 that the morphology of the interdendritic region of the as-cast and 700 °C-annealed HEA are basically the same. The formation of the *σ* phase, which is harder than the other constituent phases, can also improve the strength to a certain extent, although its fraction is low. The corresponding elongation of the 700 °C-annealed Al_0.6_CoCrFeNi HEA only retains 2.9%, which follows the traditional trade-off relationship between strength and plasticity.

As the annealing temperature is further increased, the B2 phase (thermodynamic equilibrium phase [27]) precipitates directly from the FCC dendrite according to the corresponding SADPs shown in Figure 3c and Figure 4d. However, the effect of precipitation hardening is expected to weaken gradually due to the increase in size and will give rise to the softening of the alloy compared with that of the 700 °C-annealed alloy. Correspondingly, the yield strength and tensile strength of the 900 °C-annealed alloy drops to 670 MPa and 1010 MPa, respectively, with the corresponding elongation reverting back to 6.2%. After annealing at 1100 °C, the *σ* phase fully disappeared and the interdendritic region was completely replaced by the FCC and B2 dual-phase microstructure (Figure 4). The microstructure evolution caused by phase transition and the coarsening of the B2 precipitation further weakens the strengthening effect of the alloy, resulting in a decrease in the strength. Therefore, the mechanical properties of the 1100 °C-annealed alloy return to a level close to that of the as-cast alloy, with the yield strength, tensile strength, and elongation being 492 MPa, 1007 MPa, and 19.6%, respectively.

**Figure 7:**
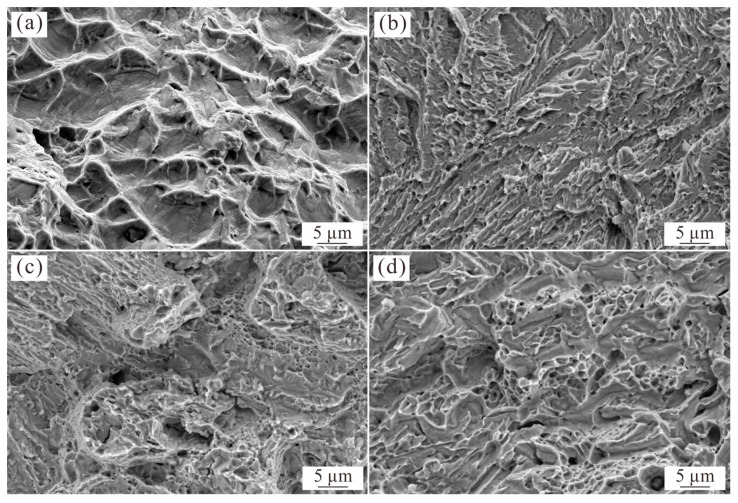
Fracture morphologies of the as-cast and annealed Al_0.6_CoCrFeNi HEAs. (**a**) as-cast; (**b**) 700 °C; (**c**) 900 °C; and (**d**) 1100 °C.

Figure 7 shows the morphology of the fracture surface of the as-cast and annealed Al_0.6_CoCrFeNi HEAs after tensile tests. The fracture surface of the as-cast Al_0.6_CoCrFeNi HEA exhibits many dimples, indicating a ductile fracture (Figure 6a). The observed large dimension of dimples corresponds to the coarse and soft FCC dendrite structure. However, the 700 °C-annealed Al_0.6_CoCrFeNi HEA exhibits typical river patterns and indicates the cleavage fracture, which is because the precipitation hardening of the nano-sized L1_2_ phase in the FCC dendrite and the dislocation movement is severely hindered. Although the precipitation formed from the FCC dendrite of the 900 °C-annealed Al_0.6_CoCrFeNi HEA converted to the B2 phase, its size is much larger than the L1_2_ phase in the 700 °C-annealed alloy (Figure 2k), and thus the effect of precipitation hardening is weakened. Therefore, the 900 °C-annealed Al_0.6_CoCrFeNi HEA exhibits mixed-mode fracture mechanism of ductile and cleavage fractures, with the presence of fine dimples. The 1100 °C-annealed Al_0.6_CoCrFeNi HEA also exhibits a mixed fracture mechanism, but is also different in its details. First, the dimension of the dimples is larger than that of the 900 °C-annealed Al_0.6_CoCrFeNi HEA, which could be attributed to the increasing size of B2 precipitates in the 1100 °C-annealed sample. Moreover, the fracture of the 1100 °C-annealed sample is flatter than that of the as-cast and 900 °C-annealed samples. This result is consistent with the corresponding FCC and B2 dual-phase microstructure (Figure 4a,b) of the 1100 °C-annealed Al_0.6_CoCrFeNi HEA consisting of the FCC dendrite region with B2 precipitation and the interdendritic region with the FCC phase in the B2 matrix.

## 4. Discussion

**Figure 8:**
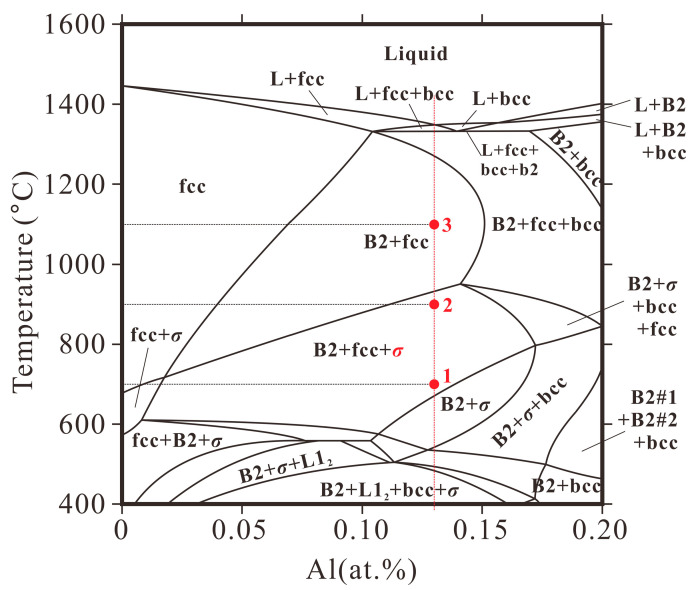
Calculated isopleth of the Al*_x_*CoCrFeNi with *x* = 0–1 [27,30]. The red dotted line shows the composition of the Al_0.6_CoCrFeNi alloy. Points 1, 2, and 3 correspond to the samples annealed at 700 °C, 900 °C, and 1100 °C.

The pseudobinary isopleth diagram of the Al*_x_*CoCrFeNi alloy system with varying Al content, reproduced from reference [27,30], is illustrated in Figure 8. The composition of the studied Al_0.6_CoCrFeNi HEA is marked with a red vertical dotted line on the diagram. The points 1, 2, and 3 correspond to the Al_0.6_CoCrFeNi HEAs annealed at 700 °C, 900 °C, and 1100 °C, respectively. However, the constituent phases of the as-cast Al_0.6_CoCrFeNi HEA (FCC + BCC + B2, Figure 2) do not seem to fit the thermodynamic prediction at low temperatures (Figure 8) due to the absence of the L1_2_ and *σ* phases. Instead, the experimental observation is consistent with the phase prediction at high temperatures (above 1300 °C). For the multi-component high-entropy alloys, the diffusion-dependent processes like crystallization, grain growth, and precipitation are expected to be slow, due to the sluggish long-range diffusion [31]. Therefore, the solidification path of the Al_0.6_CoCrFeNi melt would be for the growth of the FCC dendrite with chemical supersaturation and the residual liquid in the interdendritic region solidifies to the BCC phase, which subsequently decomposes to BCC and B2 phases. The rest of the phase transitions below 1300 °C are suppressed, resulting in the retention of the supersaturated FCC phase and disordered BCC phase in the as-cast alloy.

The thermodynamic equilibrium phases are expected to be obtained in the annealed alloys via the decomposition of the unstable phases. Therefore, the thermodynamic stable phases for this composition at 700 °C and 900 °C should be the same, namely, FCC + B2 + *σ*. The corresponding phase fractions vary as the annealing temperature is increased, which will not be discussed here. Since the *σ* phase can be obtained via the decomposition of the BCC phase in the ID region, the precipitation within the FCC dendrite is expected to be of the B2 phase. This result is consistent with the observation in the 900 °C-annealed sample, with the formation of a nano-sized rod-like B2 phase. However, the L1_2_ phase, rather than the predicted B2 phase, is formed directly from the FCC dendrite at 700 °C. The onset nucleation kinetics of precipitates (L1_2_, B2, *σ*, and BCC) were first analyzed in Al_0.3_CoCrFeNi HEA by Gwalani et al. [27]. The predicted results show that, although the B2 phase has the maximum driving force over the entire temperature range according to the calculated extended line, the nucleation barrier of the L1_2_ phase is the minimum and is lower than that of the B2 phase at temperatures below about 760 °C. These results are in line with the observations in the present paper. Despite being a metastable phase, the nano-sized L1_2_ phase is precipitated directly from the FCC phase due to the low interfacial energy between these two phases as they have the same lattice parameters (Figure 2k). The coherent characteristics of the L1_2_ precipitates and the FCC matrix have also been reported in Al_0.3_CoCrFeNi [5]. However, the nucleation barrier of the L1_2_ phase is higher than that of the B2 phase at temperatures above 760 °C. Instead, the B2 phase, possessing the maximum driving force, directly precipitated from the FCC dendrite at 900 °C. The size of the B2 precipitates, with a width of 100 nm, is larger than that of the L1_2_ phase observed at 700 °C, due to the coarsening mechanism of the precipitates with increasing annealing temperature [32]. Here, the interface of the B2 phase and the FCC matrix is not coherent due to the difference in the crystal orientation according to the high-resolution TEM analysis and lattice parameters, and the semi-coherent or incoherent characteristics are also reported in the Al_0.3_CoCrFeNi. It is clear that the constituent phases of the 900 °C-annealed Al_0.6_CoCrFeNi are consistent with the thermodynamic prediction. The FCC dendrites contain the B2 precipitates and the interdendritic region contains the B2, FCC, and *σ* phases. As the annealing temperature is increased to 1100 °C, the *σ* phase decomposes to the FCC and B2 phases and the interdendritic region also converts to an FCC-BCC dual-phase structure (Figure 4b). This result is also consistent with the thermodynamic prediction that the stable phases of the Al_0.6_CoCrFeNi at 1100 °C are FCC and B2 phases (point 3 in Figure 8).

In general, the FCC phase is soft and the FCC-structured alloys always present good plasticity yet low strength. The BCC phase is harder than the FCC phase due to the lack of slip direction, which can be used to strengthen the alloys with increasing fractions. The *σ* phase with a tetragonal structure lacks multiple slip systems, and thus is harder and brittle, which can also act as a reinforcement [33]. In addition, the disordered–ordered transition can also improve the strength of the alloy due to the reduced number of slip planes in ordered phases [34]. For the Al_0.6_CoCrFeNi HEA annealed at 700 °C, the decomposition of the disordered BCC phase begins with the formation of the softer FCC phase and the harder *σ* phase (Figure 2b,c). In some regions, the phase transition has not yet occurred. Therefore, it is assumed that the phase transition of the interdendritic region at 700 °C does not affect the mechanical properties, due to the opposite effect of the decomposed FCC and *σ* phases. Therefore, the formation of the nano-sized L1_2_ phase with a large fraction gives rise to the most significant improvement in the strength for the 700 °C-annealed Al_0.6_CoCrFeNi HEA. The corresponding fracture model converts to the cleavage fracture. The precipitation of the coarse B2 phase from the FCC dendrite, with semi-coherent or incoherent interfaces, results in the strength reduction in the alloys after annealing at 900 °C and 1100 °C. Although the formation of the hard *σ* phase in the interdendritic region would have a weak effect on improving the strength of the 700 °C-annealed and 900 °C-annealed Al_0.6_CoCrFeNi HEAs, the decomposition of the *σ* phase could have a considerable impact on softening of the 1100 °C-annealed Al_0.6_CoCrFeNi HEA. This is because the BCC + B2 structure in the interdendritic region completely converts to the soft and coarse FCC + B2 structure, wherein the fraction of the FCC phase is more than 50% in the interdendritic region (Figure 4b).

**Figure 9:**
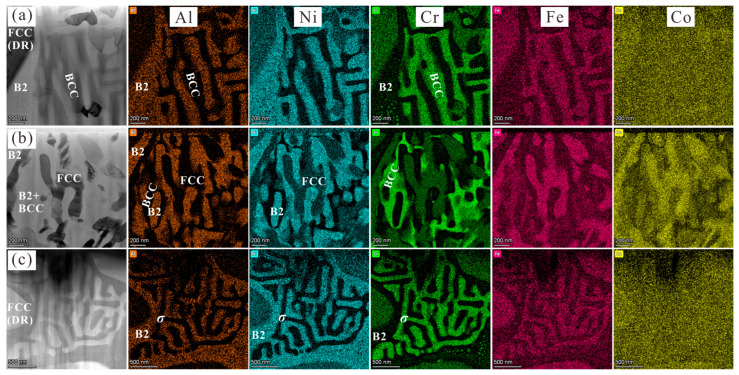
Images (**a**–**c**) show TEM micrographs corresponding to the ID-2 in Figure 1b and the ID region in Figure 2b,c, respectively. The measured elemental composition is listed on the same line in order.

Different from the co-existence of the FCC, B2, and *σ* phase obtained in the Al_0.3_CoCrFeNi HEA by means of 90% cold rolling and directly annealed at 620 °C, the disordered BCC phase in the present paper, as well as the decomposed *σ* phase at 700 °C and 900 °C, was confined to the interdendritic region. Here, there is still one problem that cannot be explained with solid evidence, namely that the co-existence of the FCC and *σ* phases, formed from the decomposition of the BCC phase, are not detected at the same time, as shown in Figure 2b,c. Even so, TEM-EDS analysis was also carried out on the interdendritic region shown in Figure 1 and Figure 2b,c, the results of which are shown in Figure 9. It is clear that, for the as-cast and 700 °C-annealed Al_0.6_CoCrFeNi HEAs, the B2 phase is rich in Al and Ni and lean in Cr and Fe. The decomposed FCC phase (Figure 9b) is rich in Cr, Fe and Co, and lean in Al and Ni, which is slightly different from that of the BCC and *σ* phases. As can be seen in Figure 9c, the decomposed *σ* phase is rich in Cr and Fe and lean in Al and Ni. According to the Cr element distribution image in Figure 9b, the remaining disordered BCC phase contains a higher composition of Cr than that of the decomposed FCC phase. Therefore, the *σ* phase might be obtained via the decomposition of the remaining BCC phase. This raises the question of whether the *σ* phase is first formed via the decomposition of the BCC phase, which might be due to the local elemental segregation during solidification (Figure 1a and Table 2) and constitutes part of future studies.

Except for the size variation in the precipitation, the potential coarsening behavior of the grain or structure originally solidified from the Al_0.6_CoCrFeNi should be considered as well, especially for the FCC dendrite. According to the SEM investigation of the as-cast and annealed Al_0.6_CoCrFeNi HEAs, it can be concluded that no obvious dendrite coarsening was found. The difference in the dendrite structure presented in Figure 1, Figure 2, Figure 3 and Figure 4 is caused by the solidification in the arc-melted casting rod, wherein the grain might present a certain orientation [4]. This is also the reason for the reflected difference in the (111)/(200) peak ratio, as shown in Figure 5.

The results also indicate the element segregation of the as-cast Al_0.6_CoCrFeNi HEA with the formation of coarse FCC dendrite, which will reduce the strengthening effect caused by the decomposition of the supersaturated FCC phase. As mentioned above, the microstructure and mechanical properties of the Al_0.6_CoCrFeNi HEA could also be tuned by deformation and subsequent recrystallization mechanisms. Recent studies also revealed that the microstructure of the as-cast HEAs can be strongly adjusted during the fabrication process with the assistance of advanced technologies, such as 3D printing [35,36], solidification assisted by directional temperature field [20], electromagnetic field [21], and high cooling rate and/or high undercooling [34,37]. The favorable microstructures could be grain refinement and weakened element segregation. Therefore, it is also an effective route to improve the mechanical properties of the HEAs related to unstable phases by means of these advanced solidification technologies combining subsequent simple annealing processes, thus avoiding the severe plastic deformation operations.

## 5. Conclusions

In conclusion, the mechanical properties of Al_0.6_CoCrFeNi HEA can be enhanced via the phase transitions of the as-solidified unstable phases using a simple heat treatment process. The maximum increase in yield strength can be achieved after annealing at 700 °C, with the increment being about 107%, which is mainly attributed to the numerous L1_2_ nano-precipitates in the FCC dendrite region. The disordered BCC phase in the interdendritic region can decomposes into FCC and *σ* phases at 700–900 °C and the *σ* phase will subsequently convert to B2 and FCC phases at 1100 °C. The softening of the 1100 °C-annealed alloy can be attributed to the coarsening B2 precipitates in the dendrite region and the soft FCC phase formed in the interdendritic region. Finally, the corresponding mechanical properties revert to values very close to those of the as-cast Al_0.6_CoCrFeNi HEA.

## Figures and Tables

**Table 1:** The measured average composition of Areas 1–3 in the as-cast Al_0.6_CoCrFeNi HEA.

		Al/at.%	Co/at.%	Cr/at.%	Fe/at.%	Ni/at.%
Nominal Composition		13.0	21.7	21.7	21.7	21.7
Area-1	Average	13.1 ± 0.1	22.3 ± 0.1	21.6 ± 0.4	21.8 ± 0.2	21.1 ± 0.4
Area-2	Average	12.9 ± 0.3	22.5 ± 0.1	21.7 ± 0.3	21.7 ± 0.2	21.2 ± 0.2
Area-3	Average	12.9 ± 0.3	22.4 ± 0.2	21.7 ± 0.3	21.8 ± 0.2	21.1 ± 0.2

**Table 2:** The measured average composition of the FCC and ID region corresponding to Areas 1–3 in Figure 1a.

		Al/at.%	Co/at.%	Cr/at.%	Fe/at.%	Ni/at.%
Area-1	FCC phase (DR)	10.6 ± 0.2	19.0 ± 0.1	24.9 ± 0.5	24.7 ± 0.7	20.7 ± 0.9
	ID region	15.5 ± 0.5	20.8 ± 0.2	23.4 ± 0.2	20.8 ± 0.3	19.6 ± 0.2
Area-2	FCC phase (DR)	11.1 ± 0.1	23.0 ± 0.2	21.4 ± 0.3	22.3 ± 0.2	22.3 ± 0.3
	ID region	15.5 ± 0.3	20.9 ± 0.2	23.5 ± 0.4	20.9 ± 0.5	19.2 ± 0.5
Area-3	FCC phase (DR)	10.8 ± 0.3	23.0 ± 0.6	21.5 ± 0.3	23.2 ± 0.4	21.3 ± 0.3
	ID region	15.7 ± 0.3	20.7 ± 0.3	23.0 ± 0.5	20.8 ± 0.3	19.8 ± 0.3

**Table 3:** EDX results of the 1100 °C-annealed Al_0.6_CoCrFeNi HEA.

	Al/at.%	Co/at.%	Cr/at.%	Fe/at.%	Ni/at.%
FCC phase (DR)	7.9 ± 0.3	24.3 ± 0.4	26.6 ± 0.2	24.6 ± 0.4	17.1 ± 0.4
B2 phase (ID)	31.7 ± 0.5	16.9 ± 0.5	9.5 ± 0.2	12.0 ± 0.4	30.1 ± 0.4

## Data Availability

Data are contained within the article.
